# A Simple Method to Optimally Select Upper-Limb Joint Angle Trajectories from Two Kinect Sensors during the Twisting Task for Posture Analysis

**DOI:** 10.3390/s22197662

**Published:** 2022-10-09

**Authors:** Pin-Ling Liu, Chien-Chi Chang, Li Li, Xu Xu

**Affiliations:** 1 Department of Industrial Engineering and Engineering Management, National Tsing Hua University, Hsinchu 300, Taiwan; 2 Edward P. Fitts Department of Industrial and Systems Engineering, North Carolina State University, Raleigh, NC 27695, USA

**Keywords:** twisting task, joint angle, data selection, regression, error trendline, Kinect sensor

## Abstract

A trunk-twisting posture is strongly associated with physical discomfort. Measurement of joint kinematics to assess physical exposure to injuries is important. However, using a single Kinect sensor to track the upper-limb joint angle trajectories during twisting tasks in the workplace is challenging due to sensor view occlusions. This study provides and validates a simple method to optimally select the upper-limb joint angle data from two Kinect sensors at different viewing angles during the twisting task, so the errors of trajectory estimation can be improved. Twelve healthy participants performed a rightward twisting task. The tracking errors of the upper-limb joint angle trajectories of two Kinect sensors during the twisting task were estimated based on concurrent data collected using a conventional motion tracking system. The error values were applied to generate the error trendlines of two Kinect sensors using third-order polynomial regressions. The intersections between two error trendlines were used to define the optimal data selection points for data integration. The finding indicates that integrating the outputs from two Kinect sensor datasets using the proposed method can be more robust than using a single sensor for upper-limb joint angle trajectory estimations during the twisting task.

## 1. Introduction

The trunk twisting posture is frequently found in occupational settings, which can be found in various industries, e.g., nursing [1,2], construction [3], and manufacturing [4]. The relationship between awkward posture (e.g., twisting) and the risk of work-related musculoskeletal disorders (WMSDs) was previously evidenced [5]. Most research has focused on the effects of the twisting movement on lumbar moments [6] or ergonomic assessment scores [4]. Upper-limb injuries are a major complaint in the workplace among janitorial workers [7], physical therapists [8], etc. According to the annual statistics report published by the Health and Safety Executive in 2021, cases in upper limbs or neck areas accounted for 45% of 470 thousand workers suffering from WMSDs [9].

An ergonomically designed working environment may help to reduce the occurrence of ergonomic issues [10]. Exposure assessment plays an important role in determining the priority of ergonomic interventions; therefore, efficient strategies of ergonomics exposure assessment that allow more accurate predictions of injury risk need to be defined [11]. Joint kinematics can be used as an ergonomics tool that identifies inappropriate working strategies to decrease the risk of injuries [12]. It is important to measure the upper-limb joint angle to assess physical exposure to upper-limb MSDs in the workplace; if it is because of the working environment, including the task design and layout arrangement, there is a requirement for improvement that should be examined, thereby promoting appropriate ergonomic interventions.

Typically, joint angles are obtained using a conventional marker-based motion tracking system (MTS); however, the need to use an array of cameras makes their implementation unrealistic in the field [13]. Correspondingly, a low-cost and markerless Kinect sensor can be an affordable 3D sensing device and provide an accessible alternative to MTSs for working posture assessment in field studies for companies that do not have the financial capacity to afford high-priced systems and for full-time ergonomists to use the assessment tools properly. Microsoft has released three different Kinect sensor versions [14]. Each one of the versions can identify the locations of a human skeleton which is composed of a specific body landmark amount [15]. The applicability of the three versions of the Kinect sensor has been studied and includes both the Kinect v2 [16] and Azure Kinect in gait analysis [17], the Kinect v2 for kinematic assessments [18], and also movement measurements for patients performing clinically functional movements [19] and wheelchair transfer tasks [20] based on Kinect v1. However, the literature is scarce on the accuracy of using Kinect sensors in monitoring upper-limb joint angle trajectories during twisting tasks based on ground truth data from an MTS.

Aimed at conducting a more efficient long-term risk assessment in industrial practice, Kinect sensors have also been used to develop semi-automatic evaluation tools based on observational ergonomic methods, such as Rapid Upper Limb Assessment (RULA) [21] or the Ovako Working Posture Assessment (OWAS) [22]. These are used to address certain limitations that exist in manual subjective observation, such as a low sampling rate and inter-/intra-rater variability. However, different accuracy levels were observed in Kinect sensors at different viewing angles [23,24]. A previous study stated that the optimal placement of the Kinect sensor is task-dependent, and the performance of kinematic measurement when using a single Kinect-based motion capture system should be examined carefully during each upper limb functional task, especially under scenarios when body occlusions existed [25]. Occlusions can reduce the accuracy of an optical system if a sensor’s field of view (FoV) is blocked [26]. The occurrence of occlusions due to surrounding objects [27] and self-occlusions [28] was reported to be a problem when assessing work tasks using the data collected via Kinect sensors. A large-scale study tested more than 500 thousand configurations, which were based on a virtual mannequin in various poses being tracked using Kinect sensors from different orientations. The results indicated that the pose estimation inaccuracy strongly increases when the occlusions are induced by some specific upper limb poses and the sensor placements [29]. A Kinect-based system, under tracking conditions and with intended occlusions, did perform worse when compared to cases without occlusions [30]. With respect to tracking upper-limb joint angle trajectories during twisting tasks in the workplace, the projection angle of a worker relative to a sensor can frequently change; further, a variety of objects (i.e., box, conveyor, or worktop) can occlude the sensor view. Therefore, it is reasonable to hypothesize that integrating multiple Kinect sensors can be more robust than using a single Kinect sensor for measuring upper-limb joint angle trajectories during twisting tasks, thereby providing effective information for performing physical work exposure assessments. On the other hand, instead of focusing on the improvement of those measurements under only one twisting condition, a method that provides a systematic procedure for relevant applications that can obtain more accurate data is worth developing.

Various technical solutions have been developed to deal with the problems of Kinect data fusion from multiple viewpoints. Moon et al. [31] have developed a human skeleton tracking system that is based on combining measurements from five extrinsically calibrated Kinect sensors by employing a Kalman filter framework. The system showed better accuracy in identifying the 3D position of body joints when compared to the results generated via a single Kinect sensor as well as a simple average method. A solution for skeletal data fusion from three Kinect sensors using algebraic operations in vector space was established for improved monitorization and analysis of the motion characteristics during physical exercise [32]. Compared with a single Kinect system, the proposed multi-Kinect system showed a 15.7% improvement in accuracy. However, the use of such techniques usually requires specific calibration procedures for multiple devices, as well as a deep knowledge of technical mathematics. Although the method itself has the possibility to be realistic in practical applications, the on-site ergonomic practitioners may not be familiar with these complex methods, thus making them inapplicable in evaluations for each of the tasks. Hence, it will be helpful for a simple but systematic method to exist that can be followed to collect data with comparable accuracy for the use of ergonomics assessments. With the help of commercial software, a previous study [26] merged two Kinect sensors’ data from two different perspectives to reduce the influence of occlusion when capturing human upper-limb motions. However, the measurement errors still increased when the participants held a box during the experiment. The second Kinect sensor does not appear to fully compensate for another sensor’s occluded view. In terms of this consideration, the question of how to select accurate data from multiple Kinect sensors at different viewing angles instead of using all raw data from the sensors to form a Kinect-based motion-tracking system is worthwhile to explore.

This study aimed to provide and validate a simple method to optimally select upper-limb joint angle data from two Kinect sensors during the twisting task using polynomial regression for ergonomic analysis. In addition, the measurement error of using Kinect sensors to monitor upper-limb joint angle trajectories when twisting was also examined.

## 2. Materials and Methods

### 2.1. Participants

Twelve healthy participants (age: mean = 23.50, SD = 1.00 years old; height: mean = 170.88, SD = 6.41 cm; mass: mean = 64.58, SD = 10.55 kg) without a history of musculoskeletal diseases were recruited and asked to perform a rightward twisting task. Informed consent was obtained from all participants involved in the study. The experimental protocol was approved by a local Institutional Review Board at National Tsing Hua University, Taiwan.

### 2.2. Procedures

**Figure 1:**
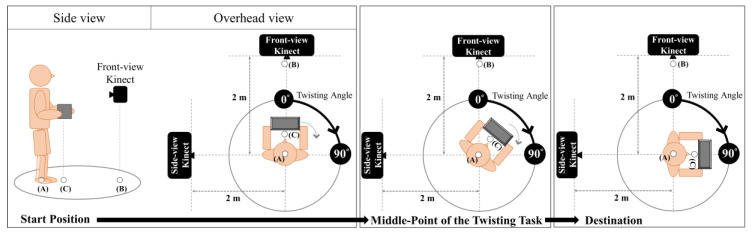
The simulated twisting task in this study. (A) is the point on the ground between the participant’s feet, (B) is the point on the ground underneath the Front-view Kinect, and (C) is the projected point on the ground from the midpoints of the participant’s two wrist locations.

A total of 32 skin markers were attached to the participants’ body landmark locations based on the “Rab Upper Extremity Model” [33] and the “Conventional Gait Model” [34,35,36,37,38,39], which can be implemented in a professional biomechanical software Visual3D (C-Motion Inc., Boyds, MD, USA) to compose a whole-body model for analysis. The participants were then asked to hold a box (36 cm × 16 cm × 17 cm) in a standing position with two hands on either side of the box. The box was intended to create occlusions with respect to Kinect sensors at different viewing angles. The participants’ upper arms were positioned perpendicular to the ground and aside from the trunk. The elbows were in a 90° flexion at the ready pose. The participants were then asked to carry the box and twist the trunk 90° to the right at their self-selected pace without moving their legs (Figure 1). Each participant performed this twisting task once.

Two Microsoft Kinect v2 sensors were placed in front of the participants (Front-view) and at the left of the participants (Side-view). The horizontal distance between each sensor and the participant was 2 m, and the heights of both sensors were 0.75 m. It should be noted that this study deliberately set up the Side-view Kinect on the left side of the participants to track the rightward twisting motion that moves away from the sensor to create a poorer tracking condition. This situation always has a chance to take place at the work site. It would be worth exploring a solution to counter such critical tracking conditions rather than under an ideal setup. The projection angles of a worker relative to a Kinect sensor in front of him/her and another Kinect sensor placed on his/her right side are complementary. The trends of the data based on these two sensors should be easier to infer in theory.

Kinect SDK 2.0 was used to develop an application for recording and outputting coordinate data of joint locations based on the Kinect skeleton model at 30 Hz. The OptiTrack motion tracking system (NaturalPoint, Inc., Corvallis, OR, USA) was used to track the reference motion data at 125 Hz. The postural data were simultaneously tracked using two Kinect sensors and the MTS.

### 2.3. Data Processing

**Figure 2:**
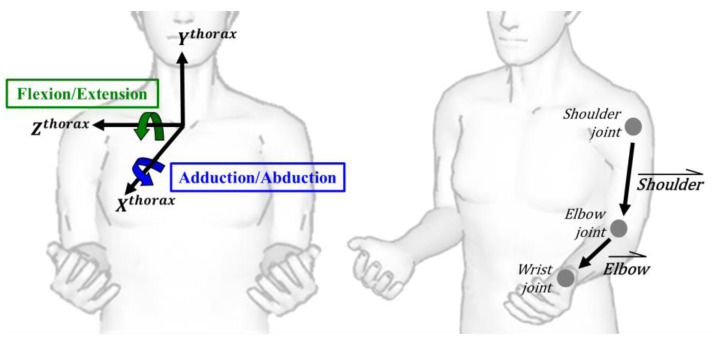
The thorax coordinate system and definition of movements.

The reference data captured through the MTS were first inputted into Visual3D to obtain the anatomical joint center locations for the following analyses. The MTS-based anatomical coordinate system of the thorax was defined based on recommendations from the International Society of Biomechanics [40]. For the Kinect-based data, the thorax coordinate system was created by referring to a previous study [24]. The adduction/abduction and flexion/extension angles of the shoulder and elbow with respect to the orientation of the thorax were calculated based on two Kinect sensors and the MTS, respectively. The adduction/abduction movement was obtained from the *x*-axis rotation, and the flexion/extension movement was obtained from the *z*-axis rotation (Figure 2).

To calculate the participant’s twisting angle, two reflective markers were placed on the ground. One was placed on the ground between each participant’s feet (A), and the other was placed on the ground and underneath the Front-view Kinect (B). The midpoints of both wrist locations were extracted from the MTS dataset and then projected onto the ground plane (C). The locations of points (A), (B), and (C) are shown in Figure 1. The twisting angle was calculated using Equation (1), where ${\vec{v}}_{AB}$ expresses the vector from point A to B and ${\vec{u}}_{AC}$ represents the vector from point A to C.

(1) $$\theta =\cos^{-1}\left(\left({\vec{v}}_{AB}\cdot {\vec{u}}_{AC}\right)/\left(\left|{\vec{v}}_{AB}\right|\left|{\vec{u}}_{AC}\right|\right)\right)$$

Reference data from the MTS were synchronized with the Kinect-based data using the network time protocol. The joint angle of the upper limbs per twisting angle was calculated based on the Front-view Kinect, the Side-view Kinect, and the MTS. The differences between the MTS-based angle and the Kinect-based angle were calculated as errors.

### 2.4. The Proposed Simple Method to Find the Optimal Data Selection Point

Polynomial regression has been extensively used in almost all topics of science since it is a tool to study the relationship between variables [41]. It seems that there is no agreement on the right order of the polynomial for the best data fitting. An overfitted complex model typically shows low bias and high variance; this shows that it can be heavily affected by the noise from the training dataset [42], and then fail to replicate in future samples [43]. To avoid a model that is too simple or overfitted, the highest polynomial order that has been used in relevant works is preferred to be used in the proposed method. The third-order polynomial regression was used to generate angular displacement of body joints in a biomechanical lifting technique analysis system [44], as it is suggested as an appropriate option to estimate muscle moments based on a limited number of input variables [45].

**Figure 3:**
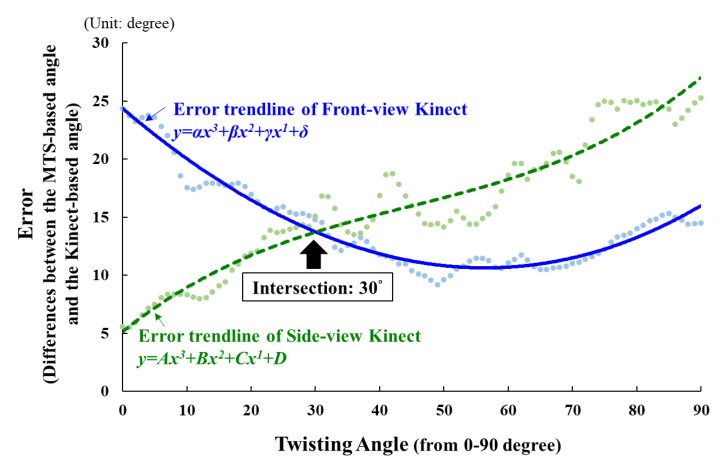
An example of our proposed method: the error trendlines of the Front-view Kinect (**—**) and the Side-view Kinect (**----**) for tracking each upper-limb joint angle trajectory are used to find the intersection point between the two tracking error trajectories. The intersection point can be defined as the optimal data selection point for switching the sensor’s viewing angle. For example, in this figure, the data of the Integration Kinect are based on the 0° to 29° duration joint angle data from the Side-view Kinect and the 30° to 90° duration data from the Front-view Kinect.

The error values in the entire twisting duration of two Kinect sensors (Front-view and Side-view) were calculated. For each Kinect sensor, the error values were fitted to generate the error trendlines of each segment’s movement using third-order polynomial regression based on the whole dataset. Regression was then used to predict the error value based on the twisting angle (per degree). Discrete errors were used to find an intersection between two error trendlines as the optimal data selection point (Figure 3). The data source to integrate the two Kinect sensors could then be defined to form the Integration Kinect. If two or more intersections existed between two trendlines, the sensor data that had an overall higher error during the entire twisting task were excluded.

### 2.5. Analysis

**Figure 4:**
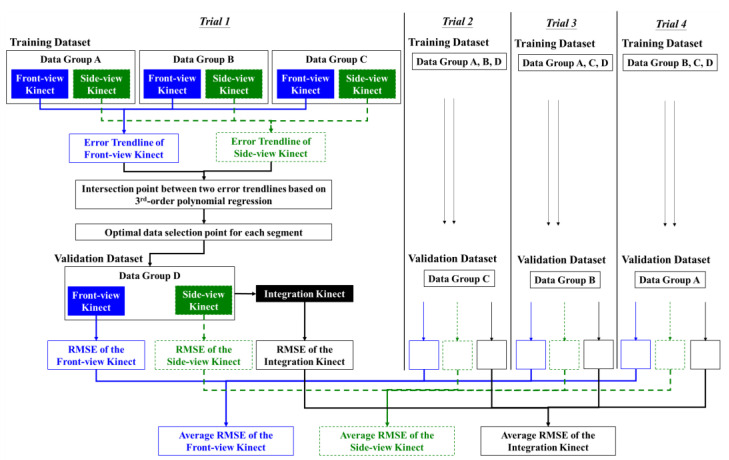
The framework to validate the proposed method.

All of the data were divided into four data groups. As shown in Figure 4, the error values collected from three of the four groups as the training dataset were applied to identify the optimal data selection points for integrating the data in each upper-limb segment’s movement based on the proposed method. The defined optimal data selection points were then used to integrate the data of the remaining group. A total of four trials were performed, and each trial alternately took one data group at a time as the validation dataset. In a trial, the RMSEs from the Front-view Kinect, the Side-view Kinect, and the Integration Kinect to estimate upper-limb joint angle trajectories were calculated based on the data collected using the MTS. The RMSEs from the four trials were then averaged. The Shapiro–Wilk test was used to check the assumption of normality. Where data were found to follow a normal distribution, a one-way analysis of variance (ANOVA) was applied, otherwise a non-parametric method (Wilcoxon signed-rank test) was conducted to check the error difference between the use of different Kinect sensors. This analysis was performed using IBM SPSS version 22.0. The significance level was set at a *p*-value < 0.05.

## 3. Results

**Figure 5:**
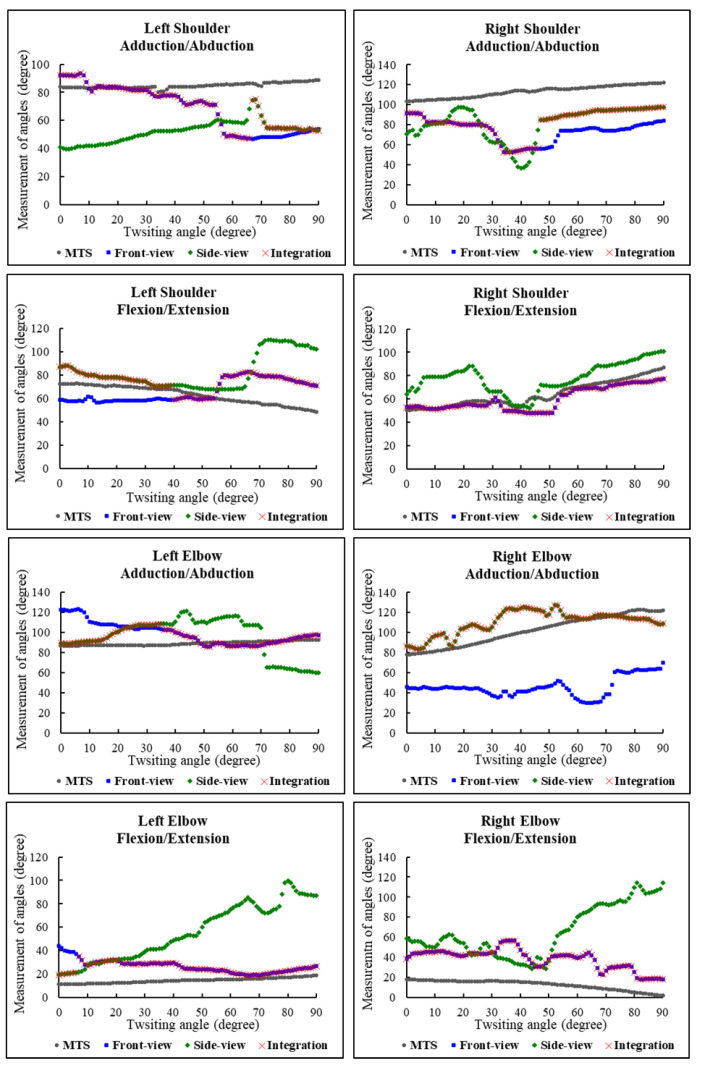
Representative example of the measured joint angle based on the Front-view Kinect, the Side-view Kinect, and the Integration Kinect with respect to the MTS. The gray circles represent the measurement obtained from the MTS, and blue squares and green diamonds indicate the data based on the Front-view Kinect, and the Side-view Kinect, respectively. The red cross sign marks are the data selected to form the Integration Kinect.

Representative examples of the upper limb joint trajectory measurement during the entire twisting task comparing the Front-view Kinect, the Side-view Kinect, and the Integration Kinect against the MTS are provided in Figure 5. Except for the errors of using the Integration Kinect in estimating the left elbow flexion/extension movement (*p*-value = 0.004), all of the other error values conformed to the assumption of a normal distribution (*p*-value > 0.05).

**Figure 6:**
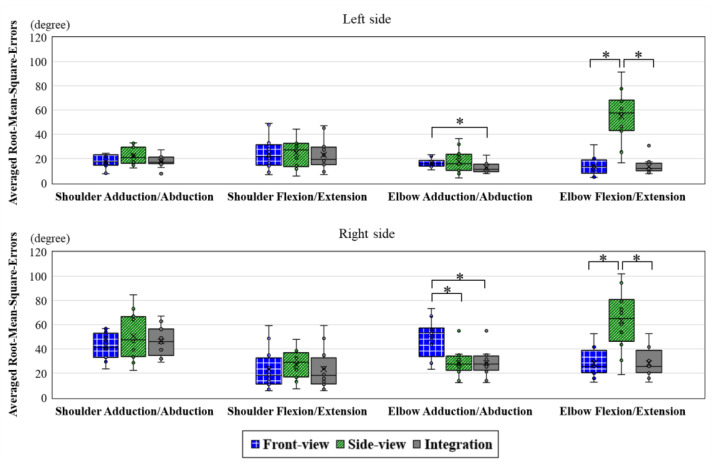
Comparison of the averaged root-mean-square error (RMSE) of the Front-view Kinect, the Side-view Kinect, and the Integration Kinect for adduction/abduction and flexion/extension movement estimates in each upper-limb segment (left shoulder/left elbow/right shoulder/right elbow). * indicates that there was a significant difference between the two datasets based on the Wilcoxon signed-rank test (*p*-value < 0.05).

The RMSEs of using the Front-view Kinect and the Side-view Kinect for tracking the twisting task were calculated based on the reference data measured via the MTS. The error values of the integrated data obtained by applying the optimal data selection method are also provided in Figure 6. The averaged RMSEs based on the four experimental trials are 26.85°, 36.18°, and 24.18° for the Front-view Kinect, the Side-view Kinect, and the Integration Kinect, respectively. The Integration Kinect produced the lowest error among them, with an overall reduction in the estimation errors with respect to the Front-view Kinect and the Side-view Kinect at around 3° and 12°, respectively.

## 4. Discussion

This study presented a simple method to optimally select the output data of two Kinect sensors for upper-limb joint angles during the twisting task. Overall, the estimates of the upper-limb joint angle trajectories based on the proposed method showed better accuracy for monitoring the twisting movement in comparison with the uses of a single Front-view Kinect or Side-view Kinect. The main hypothesis that integrating the output from two Kinect sensor datasets can be more robust than using a single Kinect sensor for upper-limb joint angle trajectory measurements during the twisting task was verified.

This study observed various levels of measurement errors for tracking different upper-limb joint angle trajectories (Figure 6). Larger errors were found at the right joint angle trajectory measurement compared with the data of the corresponding joint angles in the left segments. A previous study reported a similar result in which the estimates of the left-side shoulder joint angles were more accurate than the right-side shoulder measurements when the Kinect sensors were located to the left of the participants [24]. This finding implies that an appropriate sensor view angle is important when tracking joint angles. Another reason could be speculated that both self-occlusion and object occlusion can also influence accuracy. The identification of the right segments was interfered with by participants’ left segments, box, or/and trunk for the Front-view Kinect and Side-view Kinect during the rightward twisting task. The occlusions induced by the performed poses and the relative location of the virtual mannequins, compared with those of the Kinect sensor, were indicated to be factors that led to inaccurate data [29]. That study used a numerical mannequin with well-controlled poses to evaluate the tracking accuracy of human limb motion, with an error that increased to exceed 40° for shoulder angle measurement during a few specific body configurations when the mannequin’s body was partially occluded by other segments in the Kinect axis [27]. Another study [46] also showed that it was challenging to estimate human kinematics through Kinect sensors when occlusions were present, due to human–object interactions. By using a Kinect-based system to compute major joint angles during various tasks with/without intended occlusions, the mean error values were 13.4° and 18.3° for the tasks without intended occlusions and the tasks with intended occlusions, respectively [30]. According to Figure 6, compared with using a single Kinect sensor, using the Integration Kinect could significantly improve tracking accuracy in the elbows on the right and left sides. The use of a single Front-view Kinect would produce significantly higher errors in monitoring the adduction/abduction movement of the elbow on both sides of the body. Based on these findings, the Side-view Kinect might be considered to be a better option for this body part. However, a single Side-view Kinect showed a significantly higher error level in capturing the right and left elbow flexion/extension movements. It showed that setting up a Kinect sensor at only one location could be adequate for the needs of tracking certain segments’ movements, while it might compromise the accuracy of tracking other segments’ movements during a twisting task. When the Kinect sensors were placed at the azimuth angle of 30° and 60° on the subject’s both sides, the Kinect sensors located at the contralateral to the tracked moving arm showed a higher error in tracking the range of motion in comparison with those from the Kinect at the same side [47]. Integrating the data of upper-limb joint angle trajectories from two Kinect sensors at different viewing angles can help improve the adverse conditions for simultaneously tracking the upper limb movement of both sides. Although using the joint data based on the Kinect skeleton model to measure upper-limb joint angle trajectories still yielded errors, the proposed method in this current study did improve the outcomes in comparison with the use of either one of the Kinect sensors alone.

A previous study [26] that merged two Kinect sensors to measure upper-limb joint angles when the participants performed basic movements while handling a box also found high error values. For example, the highest RMSE was 40.7° in the flexion/extension angle measurements of the left elbow segments. In that previous study, the participants stood straight without moving their legs or bending their backs during the basic movement tasks; in contrast, the twisting task performed in this study consisted of a continuously large range of motion. Nevertheless, integrating the two Kinect sensors’ data based on the proposed method kept the error of tracking elbow flexion/extension angle trajectories below 30°, regardless of the body sides. On the other hand, compared with the RMSEs associated with using Kinect sensors to assess 3D shoulder kinematics during computer use, including typing, reading, and clicking tasks [24], slightly higher RMSEs were found in this study when using a Kinect sensor to track upper-limb joint angle trajectories in a twisting task. Wang et al. (2015) compared the accuracy of estimating joint positions during seated and standing exercises using Kinect sensors; the results showed that sitting poses generated smaller variability postures because they consisted of a greater number of static joints [23]. In addition, the occlusions created by the holding box during twisting might also contribute to higher errors. Utterly distorted skeletons with significant errors in estimating joint positions could be observed from Kinect during occluded conditions or tracked from non-frontal views [30]. In the case of this study, extremely large error values were observed when the Kinect model misidentified joint locations during twisting. The RMSE values of the proposed Integration Kinect under this condition still cannot avoid effects that result from those extreme errors. Since the Kinect skeleton model is a non-anthropometric kinematic model, higher limb length variability can be produced based on its output joint data [48]. To improve the skeletonization of the Kinect skeleton model, an additional anthropometric model could help. It is important to notice that bone lengths have been used as constraints to overcome the problems during synthesizing the inconsistency of skeletons, which are identified via duplex Kinect sensors [49]. Yet, the concern of how to incorporate other advanced techniques to reduce errors caused by misidentification of a human skeleton model, while retaining the advantages of applying the proposed method such as ease of use and no complicated calibration procedure required, is worth exploring in the future.

Researchers have continuously dedicated themselves to finding a better way to obtain more valid human movement data based on combining data from multiple Kinect sensors, such as using Kalman filtering [31] and algebraic operation [32], to perform the data fusion. This can also be performed by identifying a constrained optimization model to overcome the problems when synthesizing the inconsistency of skeletons generated by two Kinect sensors [49]. However, such advanced techniques typically incur complex procedures, requiring robust hardware or efficient software for synchronizing and fusing data, therefore making them less practical for the on-site practitioners to perform ergonomic assessments under real workspace conditions. To this end, considering that the proposed method is relatively easy to use, it could be a good option depending on the desired accuracy.

This study has several limitations. First, different orders of polynomials can create different curve fittings of the errors to identify the intersections between two trendlines. To avoid overfitting, this study used a third-order polynomial based on previous studies [44,45]. The effect of the orders of the polynomial on the results was not investigated. Second, the joint angle measurement of the upper limbs per twisting angle is static. In the current stage, the herein-reported error values may not be able to be used to directly represent the applicability of using the proposed method in some certain comprehensive kinematic analyses. The application of the proposed method to effectively acquire other measures (i.e., kinematics, tracked markers, and segment lengths) has not been examined in detail within the scope of this study. Further research is worthwhile to expand the applicability of the idea of our proposed method. Third, participants were asked to perform the twisting task at their self-selected speed. The effect of speed on such determinations was not included in the scope of this study. Lastly, the experimental postures were limited in that participants could not move their feet during the twisting task. Although different twisting postures could occur in reality and contribute to upper-limb joint angle measurement errors, controlling these variabilities allows us to understand the effects of twisting angles on the accuracy of Kinect sensors.

## 5. Conclusions

This study provides a simple regression method to optimally select upper-limb joint angle trajectory data from two Kinect sensors during the twisting task. The proposed method is not intended to replace accurate data obtained via an MTS. Rather, it can provide an alternative that is more suitable for on-site applications based on the consideration that the Kinect sensor is low-cost and markerless. When compared with manual data selection, this method allows us to apply the same posture measurements in a consistent manner, thus permitting the possibility of exposure evaluation over longer periods of time efficiently. The idea of this proposed method should be that it can be applied to identify specific optimal data switching points of joint angle trajectories for different twisting conditions.

## Data Availability

Data can be made available upon request to the authors.
